# Combined deep CNN–LSTM network-based multitasking learning architecture for noninvasive continuous blood pressure estimation using difference in ECG-PPG features

**DOI:** 10.1038/s41598-021-92997-0

**Published:** 2021-06-29

**Authors:** Da Un Jeong, Ki Moo Lim

**Affiliations:** 1 Kumoh National Institute of Technology, IT Convergence Engineering, Gumi, 39253 Republic of Korea; 2 Kumoh National Institute of Technology, Medical IT Convergence Engineering, Gumi, 39253 Republic of Korea

**Keywords:** Biomedical engineering, Health care

## Abstract

The pulse arrival time (PAT), the difference between the R-peak time of electrocardiogram (ECG) signal and the systolic peak of photoplethysmography (PPG) signal, is an indicator that enables noninvasive and continuous blood pressure estimation. However, it is difficult to accurately measure PAT from ECG and PPG signals because they have inconsistent shapes owing to patient-specific physical characteristics, pathological conditions, and movements. Accordingly, complex preprocessing is required to estimate blood pressure based on PAT. In this paper, as an alternative solution, we propose a noninvasive continuous algorithm using the difference between ECG and PPG as a new feature that can include PAT information. The proposed algorithm is a deep CNN–LSTM-based multitasking machine learning model that outputs simultaneous prediction results of systolic (SBP) and diastolic blood pressures (DBP). We used a total of 48 patients on the PhysioNet website by splitting them into 38 patients for training and 10 patients for testing. The prediction accuracies of SBP and DBP were 0.0 ± 1.6 mmHg and 0.2 ± 1.3 mmHg, respectively. Even though the proposed model was assessed with only 10 patients, this result was satisfied with three guidelines, which are the BHS, AAMI, and IEEE standards for blood pressure measurement devices.

## Introduction

There are two types of methods used to measure blood pressure: invasive and noninvasive. In the commonly used noninvasive method, blood pressure is measured through the pulse sound generated when the blood vessels in the forearm are compressed by injecting air into the cuff^1,2^. However, in the case of noninvasive blood pressure (NIBP) measurement using a cuff, blood pressure cannot be continuously measured. In the invasive blood pressure measurement method, blood pressure can be measured continuously. However, it is used only for patients with acute dysfunction failures, who are in a critical condition in an intensive care unit (ICU), and the blood pressure is measured by inserting a cannula into the artery^3^.

Many research groups have proposed a blood pressure measurement algorithm based on electrocardiography (ECG) and photoplethysmography (PPG) for noninvasive and continuous blood pressure measurements^4,5^. These were developed based on the known relationship between blood pressure and pulse arrival time (PAT). Therefore, blood pressure can be continuously estimated by continuously measuring changes in the PAT^6,7^. Proença et al. expressed the relationship between PAT and blood pressure using a nonlinear equation^8^, and Whong and Poon estimated the relationship between blood pressure, PAT, and heart rate through linear regression^9^. PAT can be measured through the time difference between the R-peak of the ECG and the systolic peak of the PPG; however, this difference is not easy to calibrate because it changes according to the physiological characteristics and pathological conditions of each individual. This may result in a decrease in accuracy or a problem with the reliability of estimating blood pressure for a completely new patient group rather than a verified patient group^10^. Meanwhile, several research groups have continuously estimated blood pressure using machine learning algorithms via features extracted from ECG and PPG signals. Chen et al. proposed a blood pressure estimation method using a genetic algorithm-mean influence value-support vector regression (GA-MIV-SVR). They extracted various features, including features related to PAT from ECG and PPG signals, and finally selected features to predict systolic blood pressure (SBP) and diastolic blood pressure (DBP) using mean influence value rankings. The obtained prediction performance satisfied the AAMI (Association for the Advanced of Medical Instrument protocols) standard (Error: 3.3 ± 5.5 mmHg for SBP and 1.2 ± 2.0 mmHg for DBP)^4^. Furthermore, Sharifi et al. proposed a multiadaptive regression spline (MARS) method based on ECG and PPG signals. They predicted SBP, DBP, and mean blood pressure with high predictive accuracies of − 0.3 ± 9.1 mmHg, − 0.1 ± 5.2 mmHg, and − 0.2 ± 4.6 mmHg, respectively^11^. Kachuee et al. extracted the heart rate, PPG features, and PAT features from ECG and PPG through feature engineering and used them to continuously estimate blood pressure, considering changes in PAT according to individual physiology^5^. They showed that DBP can be accurately estimated using a support vector machine method.

The aforementioned studies estimated blood pressure using features extracted by conducting a complex feature engineering process. In this paper, we used the morphological differences of ECG and PPG signal as a novel feature that includes information on PAT and avoids the complex preprocessing task. Furthermore, we propose an artificial neural network algorithm capable of continuously and noninvasively estimating blood pressure based on these morphological differences between ECG and PPG signals. The previous machine learning methods for blood pressure estimation separately trained the models for both SBP and DBP. However, the proposed algorithm is a combined deep CNN–LSTM network-based multitasking learning architecture model that can predict SBP and DBP simultaneously by considering the morphological features of the ECG and PPG signals, along with temporal features.

## Results

**Figure 1:**
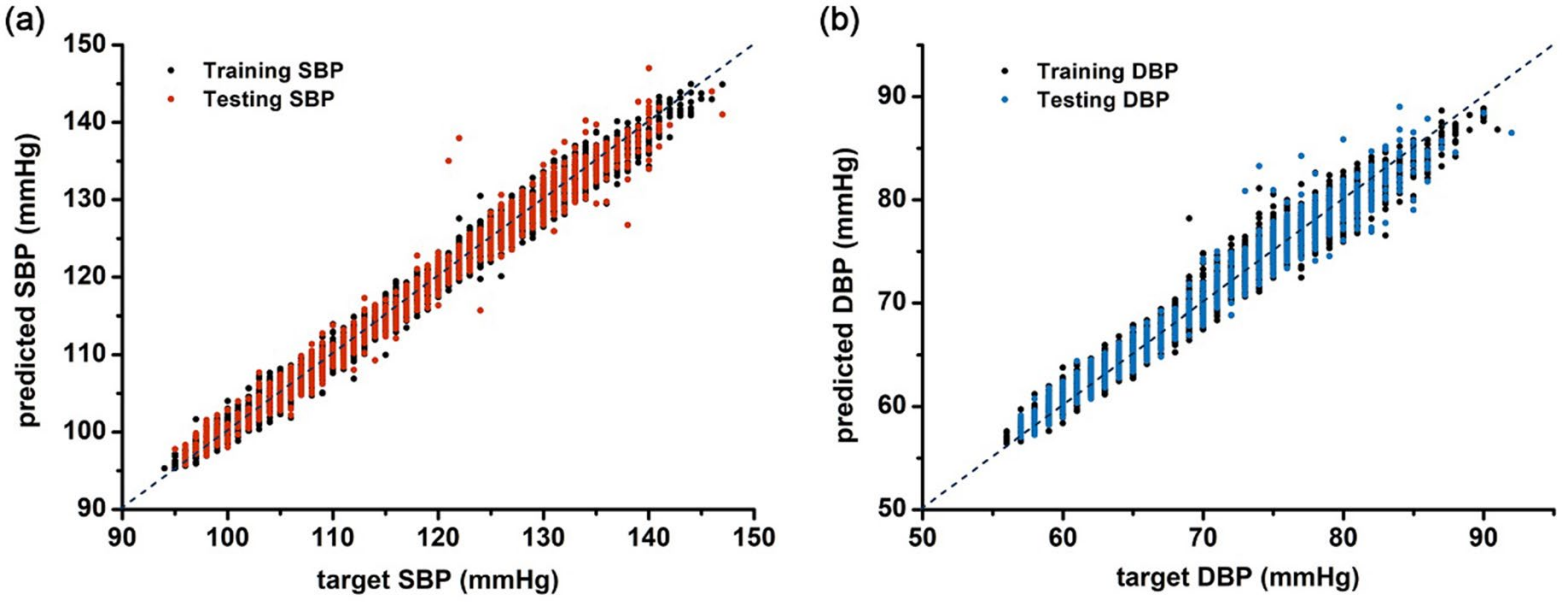
Prediction performance of the proposed model. (**A**; training and test systolic blood pressure, **B**; training and test diastolic blood pressure); For training and testing the proposed model, we used a publicly available dataset, PhysioNet’s original Multi-parameter Intelligent Monitoring for Intensive Care (MIMIC) database, which can be found here; https://www.physionet.org/content/mimicdb/1.0.0/.

The SBP and DBP used as the correct answers for supervised learning of the proposed model were 119.2 (94–147) mmHg and 70.8 (56–92) mmHg on average, respectively (Supplementary Fig. S1). We evaluated the accuracy of the blood pressure predicted by the proposed model using the determination coefficient (R^2^) and the mean squared error, which are the indicators used to evaluate the performance of the regression model (Fig. 1). The predicted accuracy of SBP was higher than that of DBP; the R^2^ values of the predicted SBP and the predicted DBP were 0.980 (p-value < 0.05, Fig. 1A) and 0.967 (p-value < 0.05, Fig. 1B), respectively. Furthermore, the adjusted R^2^ values were 0.979 and 0.966 for SBP and DBP, respectively. Accordingly, the mean squared errors of SBP and DBP were 2.7 mmHg^2^ and 1.8 mmHg^2^, respectively.

**Figure 2:**
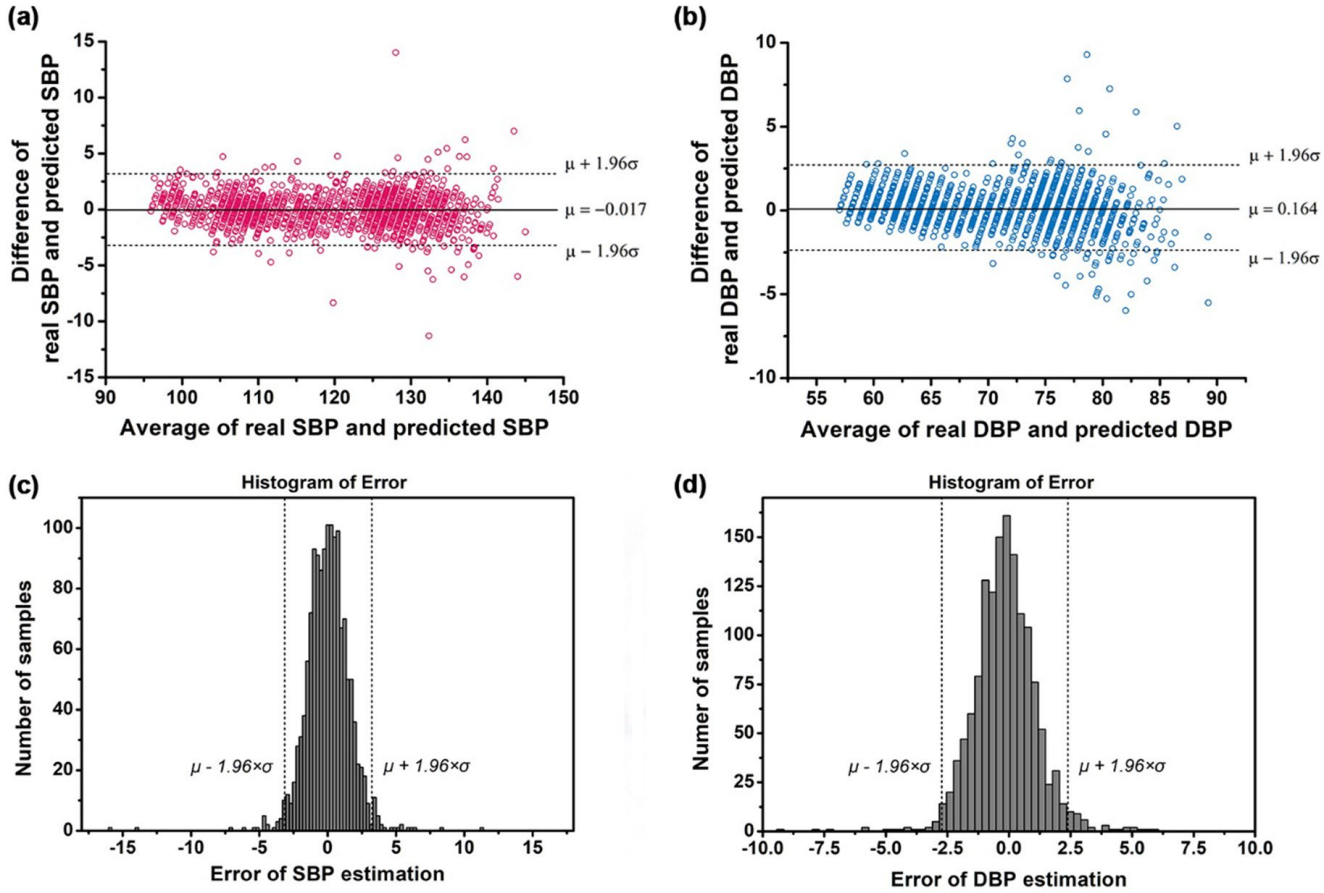
Bland–Altman plots and Error distributions of the proposed model. (**A**,**B**) Bland–Altman plots (**A**; systolic blood pressure, **B**; diastolic blood pressure). (**C**,**D**) Error histogram of predicted blood pressures (**C**; systolic blood pressure, **D**; diastolic blood pressures).

Figure 2 shows the Bland–Altman plot and the error distribution of the actual blood pressure (true value) and the predicted blood pressure to evaluate the accuracy and precision of the blood pressure predicted by the proposed model. Within the error range of ± 5 mmHg, the cumulative percentages in the predicted values of SBP and DBP were 99.4% and 99.6%, respectively (Fig. 2A,B, and Table 1). The cumulative percentage curve over the difference of real BP and predicted BP is shown in Supplementary Fig. S3. These results corresponded to Grade A according to the British Hypertension Standard (BHS), a blood pressure monitor certification standard^12^. The precision of the estimated blood pressure was confirmed using the error histogram shown in Fig. 2C,D. The errors between the estimated and target blood pressure were normally distributed at approximately 0 mmHg in both SBP and DBP. By conducting the Durbin–Watson test to verify the autocorrelation between the observed values and the predicted values of the proposed model, it was confirmed that independence of the SBP and DBP errors was satisfied (d-statics = 1.97 for SBP and 1.99 for DBP). The mean difference of SBP and DBP were 0.02 mmHg and 0.2 mmHg, respectively, and the standard deviations of the errors were 1.6 mmHg and 1.3 mmHg, respectively, which passed the AAMI standard (Tables 1 and 2)^13^. The resulting 95% confidence intervals of the predicted SBP and DBP prediction errors were (-3.2 mmHg, 3.2 mmHg) and (-2.7 mmHg, 2.4 mmHg), respectively. Besides, the mean absolute difference (MAD) was 1.2 for SBP and 1.0 for DBP, which was satisfied with the A grade of IEEE standard (Tables 1 and 2)^14^.

**Table 1:** Assessment results through three guidelines of IEEE, AAMI, and BHS standards.

Assessment standards	IEEE standard		AAMI standards		BHS guidelines		
	MAD (≤ 4 mmHg)	MAPD (%)	MD (< 5 mmHg)	SD (< 8 mmHg)	CP_5_ (> 60%)	CP_10_ (> 85%)	CP_15_ (> 95%)
**Duration = 6000 samples**							
SBP	1.2	1.0	− 0.02	1.6	99.4	99.9	100.0
DBP	1.0	1.33	0.2	1.3	99.6	100.0	100.0
**Different time interval**							
Duration = 6000 samples							
SBP	3.2	2.9	0.03	4.3	79.9	97.0	99.5
DBP	1.4	2.5	0.01	2.1	96.1	99.9	100.0
**Duration = from 10,000 to 100,000 samples**							
SBP	3.5	3.2	0.5	4.5	76.6	96.9	99.4
DBP	1.8	3.3	0.3	2.4	95.2	99.9	100.0

*MAD* mean absolute difference, *MAPD* mean absolute percentage difference, *MD* mean difference, *SD* the standard deviation of difference, *CP*_*n*_ cumulative percentage within a difference of n mmHg; *SBP* systolic blood pressure, *DBP* diastolic blood pressure.

**Table 2:** Prediction performance comparison.

Model		Error (mmHg)		BHS standard	AAMI standard	IEEE standard
		MAD	SD			
Chen et al.^4^	SBP	3.27	5.52	A	–	–
	DBP	1.16	1.97	A	–	–
Sharifi et al.^11^	SBP	0.29	9.1	–	–	–
	DBP	0.09	5.21	–	–	–
Kachuee et al.^5^	SBP	12.38	16.17	–	–	–
	DBP	6.34	8.45	B		–
Proposed model	SBP	1.2	1.6	A	Pass	A
	DBP	1.0	1.3	A	Pass	A

*MAD* mean absolute difference, *SD* the standard deviation of difference, *SBP* systolic blood pressure, *DBP* diastolic blood pressure.

## Discussion

In this study, we developed an NIBP algorithm using a combined deep CNN–LSTM network-based multitasking learning architecture. The combined deep CNN–LSTM model was constructed based on the LSTM–CNN model of Xia et al.^15^ to extract morphological and temporal features from the signal difference between ECG and PPG.

The proposed model estimated SBP and DBP using the signal difference between the ECG and PPG signals as input. The R-peak of the ECG refers to the electrical excitation time before the heart contracts^16^, and the systolic peak of PPG denotes the time until the pulse caused by heart contraction reaches the peripheral end^17^. If these two signals are connected sequentially and passed through CNN architecture, the time interval based on the R-peak of ECG and the systolic peak of PPG might be disappeared. Furthermore, the connection point of two signals can be extracted as the CNN features. Accordingly, we used the difference between the ECG and PPG signals that includes information on the electromechanical delay, which is the time delay of electrical excitation and mechanical contraction of the heart, as well as PAT (Supplementary Fig. S2).The output layer of the proposed model used a linear function^18^ to predict the SBP and DBP using a linear regression model. Next, the R^2^ value was used to measure the degree to which the estimated linear model fits the given data. This refers to the proportion of the variation in the dependent variable that can be explained using the applied model^19^. In general, the accuracy may improve as the number of independent variables in the regression model increases, but the actual data may not be properly predicted because of overfitting to the training data^20^. Therefore, the adjusted R^2^ was calculated to prevent overfitting or overestimation of the prediction accuracy of the proposed model by adding a penalty according to the effects of the added independent variables on SBP and DBP^19, 20^.

The database used in this study was obtained from ICU patients who needed intensive care and continuous monitoring. Each data point may change the shape of the signal according to the patient’s condition at the time of measurement, even if it is from the same patient. The combined deep CNN–LSTM architecture model can extract features, including both the continuous characteristics of the signal over time and the morphological characteristics of the input signal sequence. Accordingly, the predicted blood pressure was of high accuracy, but the error increased as the blood pressure increased (Figs. 1 and 2). This happened due to three reasons: First, because the average age group of the patients for whom the data were acquired was 65 years old. A sudden change in blood pressure was observed in elderly people, which created a reliability problem in the true blood pressure label used to train the supervised learning model. Second, the signal used included not only the patient’s motion artifacts that can be removed through preprocessing but also morphological changes due to certain diseases that are difficult to remove through preprocessing. For example, 10 patients had congestive heart failure (CHF), which was mainly associated with left ventricular dysfunction. The ECG of the CHF patient showed abnormalities in the wave of QT interval, which was corresponded to the previous research^21^. Besides, they had also pulmonary edema, and their PPG signals showed very unstable shapes. In these cases, In the case of these patient signals, even after pretreatment, some intervals were still unclear. Finally, it was predicted that the proposed model could not fully learn the signal characteristics of these factors. These problems were also observed in preceding studies; however, it can be seen that the error rate obtained through our proposed model is lower than that in previous studies (Table 2)^4, 5, 11^.

We compared the prediction accuracy of our proposed model with three models of previous studies^4, 5, 11^. Even though the same MIMIC database was used, the version was different. Chen et al. used the MIMIC III match subset^4^, and Kachuee et al.^5^ and Sharifi et al.^11^ used MIMIC III and MIMIC II datasets, respectively. The dataset used in this study is the original MIMIC database. In the description of Physionet, it is mentioned that the MIMIC II and MIMIC III databases are many times the size of the original MIMIC database, and they have the only advantage to include 125 "peak-picked" samples per second with 8- or 10-bit precision and ± 6 ms jitter. Therefore, it is necessary to objectively evaluate the model performance using the same dataset to be verified.

For the supervised learning of the proposed model, we used the detected SBP and DBP from the arterial blood pressure (ABP) signals in the MIMIC database. The ABP signal is invasively obtained through a catheter or cannula inserted into a blood vessel, which is mainly used to monitor the BP of patients in the ICU patient. That is, the proposed model was trained using the value of invasive BP to estimate the accurate BP non-invasively, which is closer to the value of invasive BP. In addition, since the proposed method does not have reinforcement learning, it does not receive feedback or provide additional learning via cuff BP during actual use. In other words, invasive BP and non-invasive BP are not used for learning together. Therefore, the estimated BP through the proposed model is the invasive BP, and it will not necessarily be the same as the measure BP through the standard cuff-based device.

For training and testing the proposed model, we randomly extracted the dataset during 48 s from each patient. For assessment of overfitting due to the small sample size, we extracted the same size of dataset during other 48 s in a different time interval and the longer dataset during the time interval of the 80 s to 800 s. The performance of the proposed model using these two datasets in the different time intervals was evaluated based on the three standards for BP measurement (Table 1). The accuracy of predicted BP was a little bit decreased, but it still showed great performance corresponding to Grade A. The prediction results of SBP and DBP in a different time interval were shown in Supplementary Figs. S4–S6.

Most previous studies have trained and used individual models to predict SBP and DBP. Besides, other studies developed the CNN-LSTM models to predict SBP and DBP, which separately trained the CNN-LSTM model for estimation of each BP value^15^. The estimation algorithms of BP using PAT are based on the relationship between the pulse pressure and PAT. The pulse pressure is controlled by SBP and DBP. Therefore, to precisely predict BP, a model need to be trained with consideration of SBP and DBP simultaneously. The combined deep CNN–LSTM architecture-based multitasking model developed in this study outputs both SBP and DBP simultaneously using a single model. This can lead to better performance compared to that when training separately for each factor because the common representative factors of SBP and DBP are extracted while the ECP and PPG difference signals pass through a shared layer^22,23^. It was shown that the prediction performance of the proposed model is greater than that of the models developed in previous studies.

The error histograms of the proposed model showed a normal distribution form, and it was statistically confirmed that the mean errors converged close to zero and satisfied the normality (Fig. 2C,D). In general, regression models of time-series data, such as blood pressure, may correlate with each error value^24^. This is called autocorrelation, and a regression model with autocorrelation in which the error of the predicted value of the model is affected by the error of adjacent observation values may not be completely reliable. Therefore, the Durbin–Watson test was performed to test the autocorrelation between the observed values and the predicted values of the proposed model, and it was confirmed that the independence of error was satisfied through the d-statistics adjacent to 2 for both prediction errors of SBP and DBP^24,25^.

The BHS guideline evaluates the accuracy of the sphygmomanometer’s prediction of SBP in four grades from A to D according to the cumulative percentage of the predicted SBP within 5, 10, and 15 mmHg of the errors. Here, A is the most accurate, and the accuracy decreases toward D^12^. Besides, the AAMI guidelines require that the average difference between the true and predicted values should be less than 5 mmHg, and the standard deviation for 85% of the true values should be less than 8 mmHg^13^. In the validation on 10 patients, the model proposed achieved an accuracy that satisfied both of these guidelines. These standards are for the performance evaluation of the upper-arm cuff measuring BP. Therefore, we validated the proposed model using the IEEE standard, which is suitable to evaluate the wearable and cuffless BP measurement device. The IEEE standard evaluates the performance using MAD accuracy, in which if MAD is over 4 mmHg, it would get the A grade. This standard proposes to report the performance of the BP measured for each patient before and after the calibration^14^. However, the BP estimation using a neural network does not need to calibrate according to the subject, because the neural network can extract the machine learning features with consideration of individual characteristics according to the subjects such as weight, age, gender, and so on^4,26^. Solely, it might need the calibration if it estimates BP during the long-term of 6–12 months^11^.

There are several limitations to our proposed model. First, the accuracy of long-term monitoring was not verified. For LSTM models, the accuracy may vary depending on the measurement time of the data used^27^. Accordingly, it is not known whether the accuracy of our proposed model decreases or increases when estimating blood pressure using long-term data from weeks to months. Second, the generalizability of the model was not verified. In this study, the data of ICU patients from PhysioNet were used. Although the signals were simultaneously measured, a missing signal or a signal of poor quality was removed from the dataset. For example, in some PPG signals, any fluctuated waves were not observed during the randomly extracted interval, and in some ABP signals, meaningless values such as − 2,147,483,648 or the zero states were observed. Accordingly, the number of samples used for verification of the proposed model was 7400, and the number of patients was 10, and thus the applicability of the model to many patients has not been verified. All guidelines for evaluating the accuracy of BP measurements require a minimum number of subjects that must be verified through the models or devices. The assessment guidelines we used also required a minimum number of subjects; BHS guidelines and AAMI standard require the report for over 85 subjects, and IEEE standards require over a total of 45 subjects in all phases. Even though the result we have shown in this study was satisfied with three of the guidelines, it might be due to the small number of patients. In other words, when our proposed model is tested for over 85 patients, the results could be different. Therefore, our proposed model needs to be assessed by more subjects. In conclusion, if the generalizability of the model is validated and optimized through more data, it can be applied to medical devices requiring long-term monitoring, including patient monitors and implantable cardiac devices such as the Holter ECG monitoring system. Especially, the proposed simpler algorithm would be more utilized when applied to a mobile system.

## Methods

### Dataset

In this study, we used ECG, PPG, and ABP signals measured simultaneously in PhsioNet’s Multi-parameter Intelligent Monitoring for Intensive Care (MIMIC) Database^28,29^. Whole signals were measured from ICU patients, and especially, ABP signals were obtained through invasive methods. Among the data of 57 patients (36 males and 21 females), the data that did not include ECG, PPG, and ABP, or data containing missing signals were not used. Finally, the ECG, PPG, and ABP data of 48 patients (30 males and 18 females) receiving intensive care were used for prediction, and the average age of the patients was 69.9 (21–92) years. Patients had one of 13 diseases, including bleeding, respiratory failure, congestive heart failure/pulmonary edema, brain injury, sepsis, angina, postoperative valve, postoperative coronary artery bypass graft, cord compression, trauma, renal failure, myocardial infarction, and cardiogenic shock. Each signal was acquired with a sampling frequency of 125 Hz for different recording times, but the average recording time was 42.7 (10.5–77.4) h.

### Preprocess

Simultaneously measured signals collected from the PhysioNet database were randomly extracted from each patient to obtain 6000 samples (48 s). Some extracted signals have zero values or unusable values, which are meaningless values such as − 24,975,832 or do not change according to time. These signals were removed or interpolated based on adjacent data points. To remove motion artifacts and solve the baseline wandering problem, a bandpass filter of 2 Hz to 20 Hz was applied to the ECG signal, and a bandpass filter of 0.5 Hz to 20 Hz was applied to the PPG signal. After extracting the R-peak from the filtered ECG, R(n-1) − R(*n* + 1) sequences were generated from the ECG, PPG, and ABP signals based on the detected R-peak (*n*) of the ECG to include two cycles. Zero padding was applied based on the maximum R–R sequence to compensate for the difference in the data length of the generated R–R sequence due to the change in the R–R interval according to time.

The generated ECG and PPG sequences were transformed such that the maximum and minimum were + 1 and − 1, respectively, using the MinMax scaling technique. Next, the difference between the converted ECG and PPG signals was calculated and used as the input to the combined deep CNN–LSTM architecture-based multitasking model. Besides, after extracting the peaks and inverse peaks from the ABP sequence, the average values of each peak were calculated and used as target values for SBP and DBP.

### Model structures

**Figure 3:**
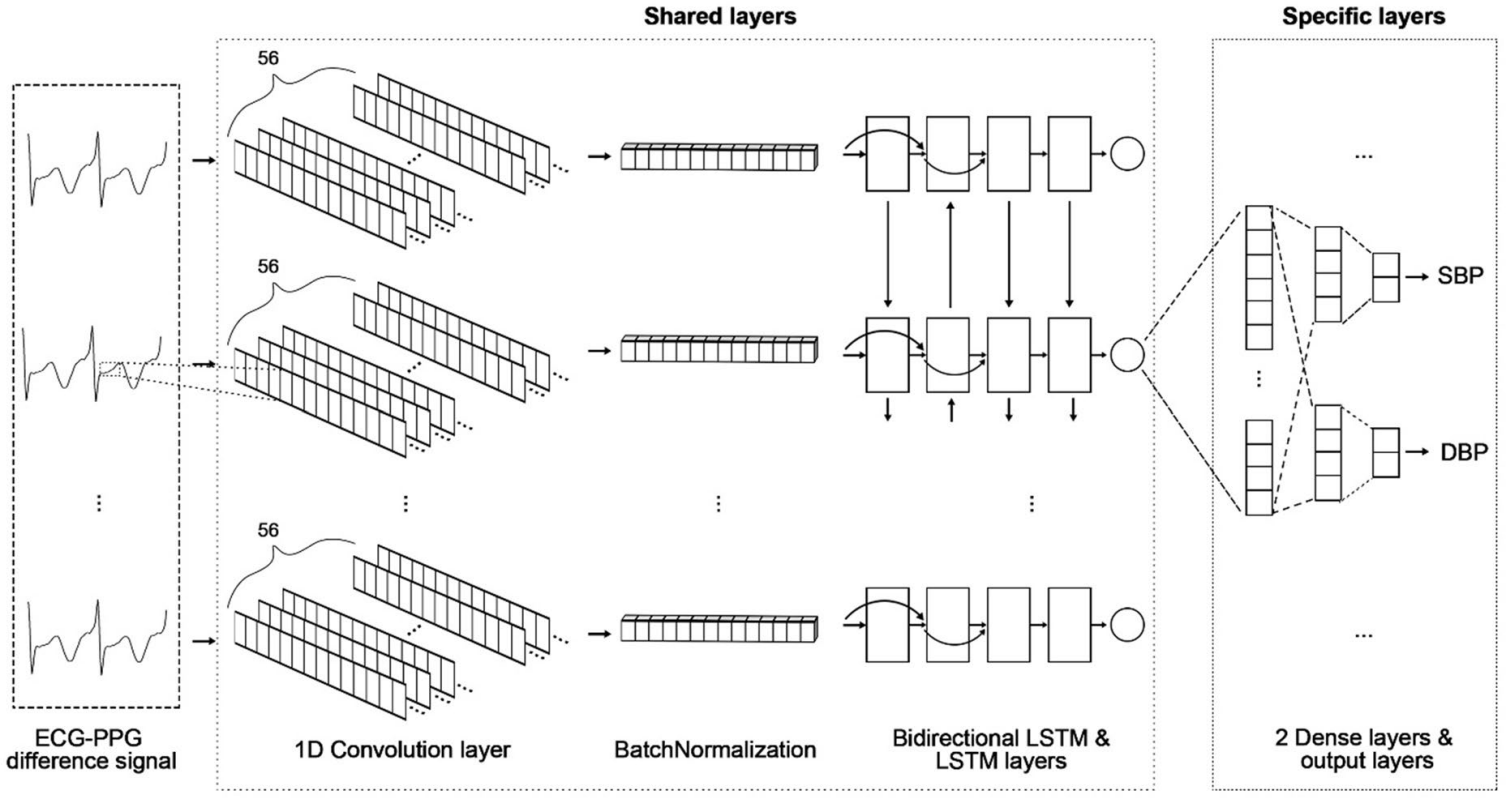
Proposed model architecture.

The proposed model consisted of a shared layer to extract morphological and temporal features from the signal difference between ECG and PPG, and a specific layer to predict SBP and DBP. The shared layer consisted of one CNN layer for morphological features and three LSTM layers for temporal features. They were connected through a batch normalization layer to prevent overfitting. The CNN layer was composed of 56 kernels of size 10, and a rectified linear unit (ReLU)^30^ was used as an activation function. Furthermore, L2 regularization was applied to improve the generalization performance of the proposed model. The three LSTM layers consisted of one bidirectional LSTM with 28 neurons and two unidirectional LSTMs connected to specific layers through a global average pooling layer. Each specific layer of SBP and DBP consisted of two fully connected layers and an output layer. The number of neurons in the fully connected layers was 28 and 16, respectively, and both activation functions were ReLU. Finally, we used a linear function^18^ as the activation function of the output layer for SBP and DBP (Fig. 3). The input layer's shape was (None, 250, 1), and the output shapes for SBP and DBP were (None, 1). Accordingly, the total number of parameters is 38,370, which has the trainable parameters of 38,258 and the non-trainable parameters of 112 (Supplementary Table S1).

### Model train and evaluation

Eighty percent of the total dataset was used to train the model and 20% to evaluate the model performance. Furthermore, 10% of the training data was used for validation to prevent the model from overfitting the training dataset. The mean squared error was used as the error function for the output result, and Adam (Adaptive Moment Estimation)^31^ with a learning rate of 0.01 and a decay rate of 0.00002 was used as the model optimization function. The model was trained 1000 times with a batch size of 28; however, the optimal model with hyperparameters was selected as the final model through early stopping.

The accuracy of SBP and DBP predicted by the proposed model was evaluated through the root mean squared error and the mean absolute error^32^, along with the value of R^2^ and mean squared error^18,19^ used in the performance evaluation of the regression model. The prediction performance was verified using the BHS^12^ and AAMI^13^ standards, which are the blood pressure monitor certification standards for upper-arm cuff validation^12,13^. Besides, the possibility of grafting the proposed model into medical devices was assessed using IEEE standard, which is the guideline for evaluation of the wearable, cuffless blood pressure monitoring devices^14^. Even though these three standards require the minimum number of patients for assessment, we didn’t follow them strictly; we validated the proposed model to only 10 patients (see the “Discussion’ section).

## Supplementary Information

Supplementary Information.

## Data Availability

In this study, publicly available datasets were used to analyze. This data can be found here: https://www.physionet.org/content/mimicdb/1.0.0/.
