# Supplementary material for: Combined deep CNN–LSTM network-based multitasking learning architecture for noninvasive continuous blood pressure estimation using difference in ECG-PPG features
Source: Sci Rep. 2021 Jun 29;11:13539. doi: 10.1038/s41598-021-92997-0 (PMC8242087; doi:10.1038/s41598-021-92997-0)
Supplement: Supplementary file 1 — Supplementary Information. [file 41598_2021_92997_MOESM1_ESM.pdf]

Combined Deep CNN–LSTM Network-based Multitasking Learning Architecture for Noninvasive Continuous Blood Pressure Estimation using Difference in ECG-PPG Features

Da Un Jeong[1], Ki Moo Lim[1, 2*]

[1]Kumoh National Institute of Technology, IT convergence engineering, Gumi, 39253, Republic of Korea.
[2]Kumoh National Institute of Technology, Medical IT convergence engineering, Gumi, 39253, Republic of Korea.
+82-54-478-7780
dawny6960@kumoh.ac.kr
*kmlim@kumoh.ac.kr

Supplementary Figures

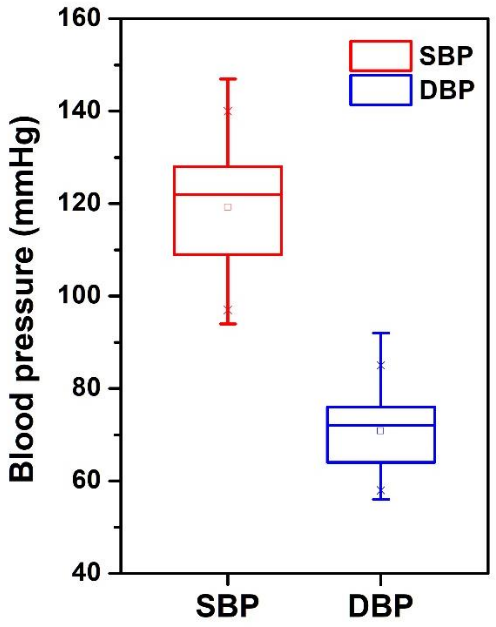

Supplementary Figure S 1. Boxplots of systolic blood pressure (SBP) and diastolic blood pressure (DBP).

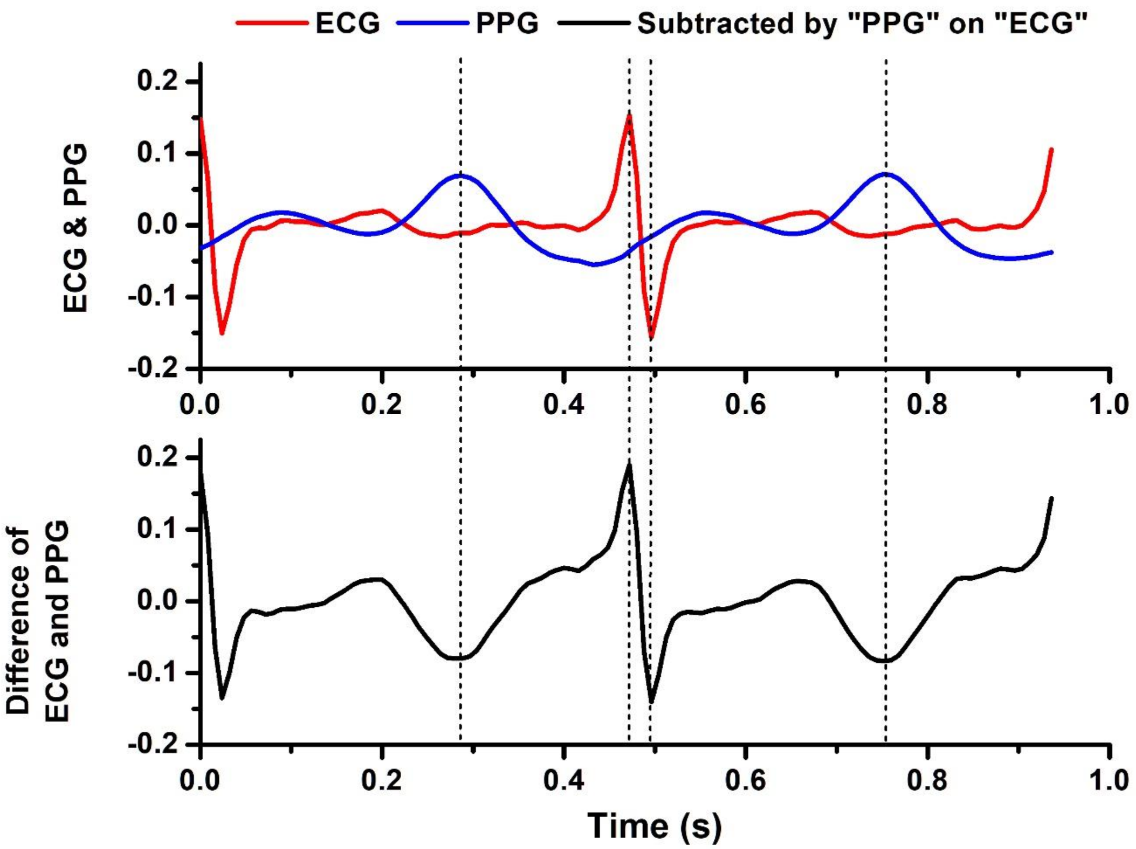

Supplementary Figure S 2. Example of signal difference between ECG and PPG.

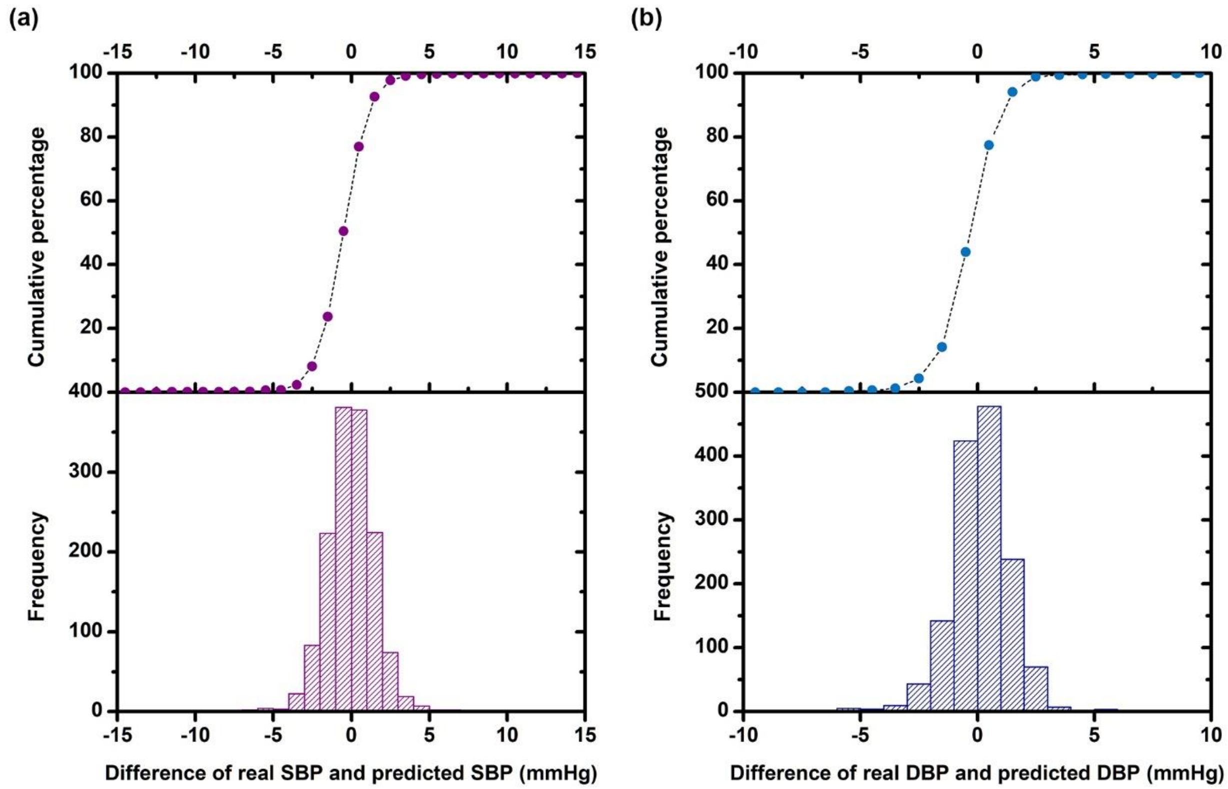

Supplementary Figure S 3. Cumulative percentage curve for BHS guideline; BP dataset was extracted during other 48 s from each patient.

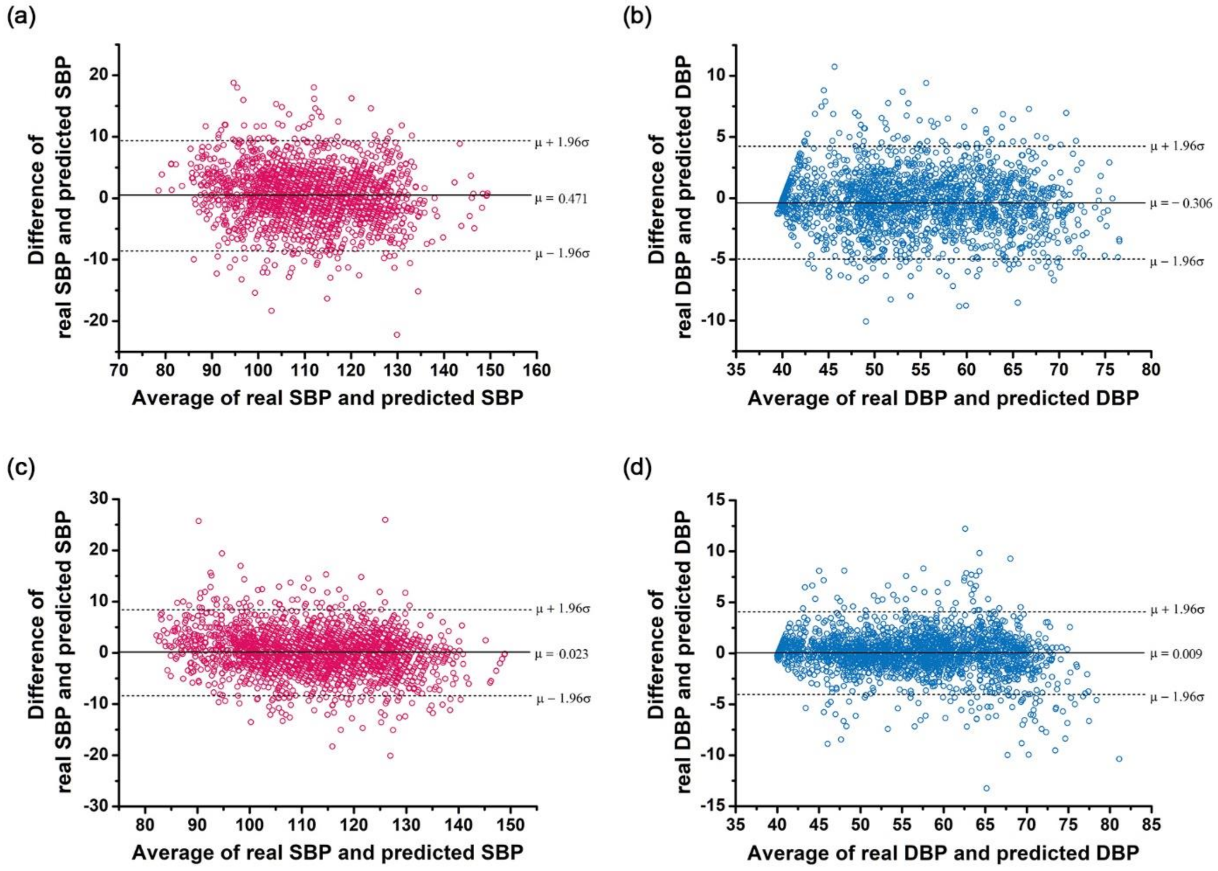

Supplementary Figure S 4. Bland-Altman plot of predicted SBP and DBP in the different time intervals.

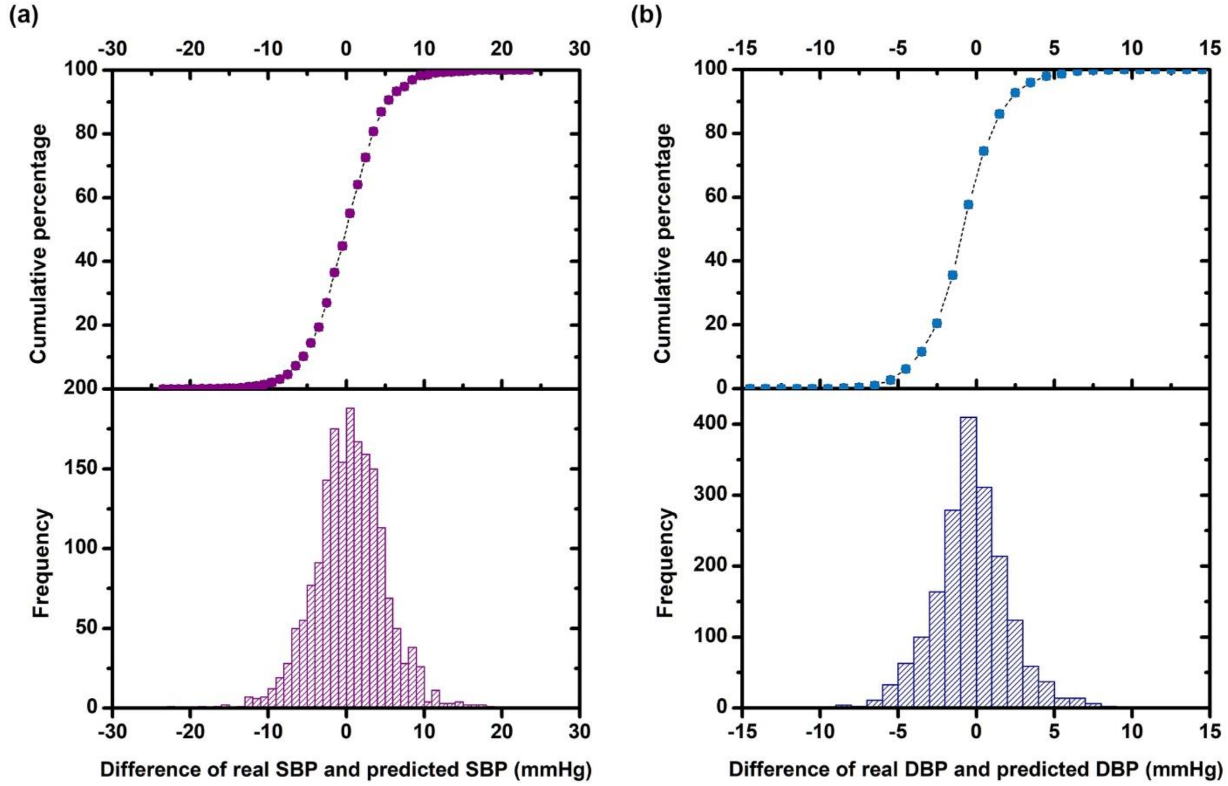

Supplementary Figure S 5. Cumulative percentage curve for BHS guideline in the different time interval; BP dataset was extracted during other 48 s from each patient.

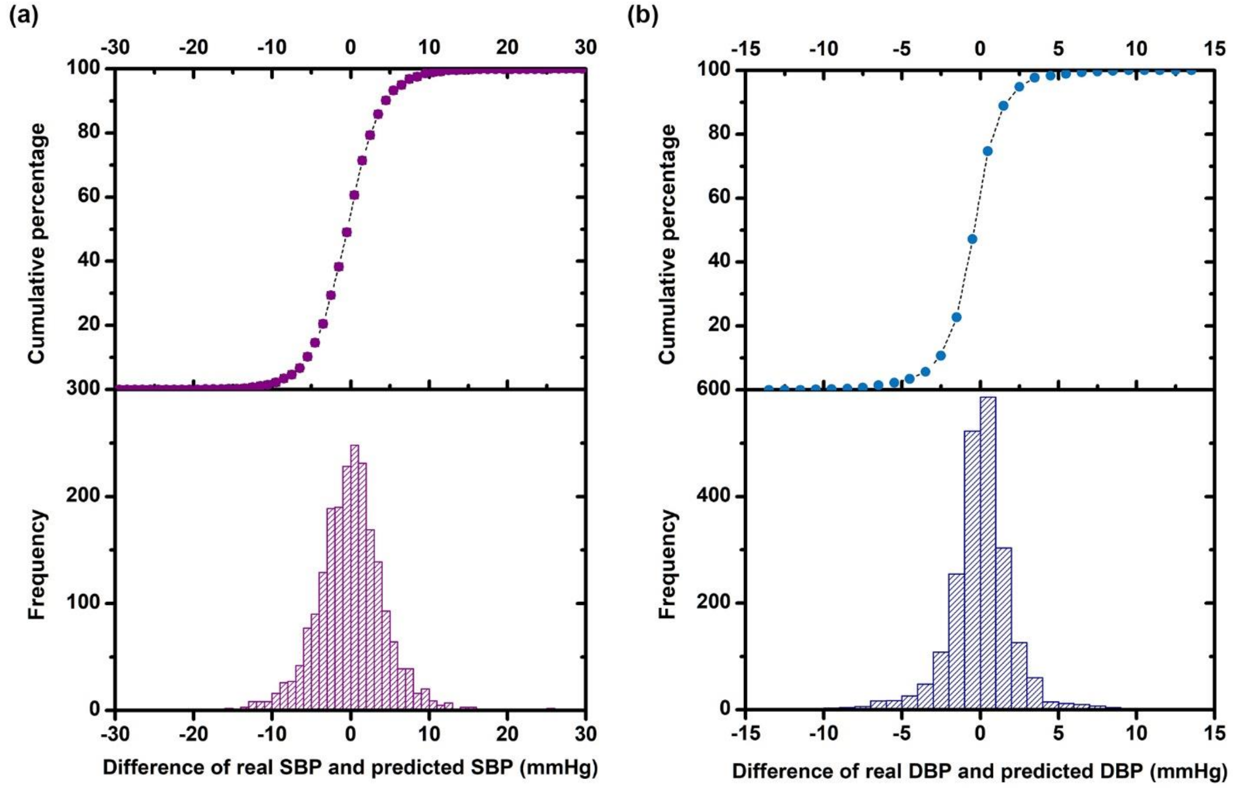

Supplementary Figure S 6. Cumulative percentage curve for BHS guideline in the longer time interval and; BP dataset was extracted during 80 s to 800s from each patient.

Supplementary Tables

Supplementary Table S 1. Parameters of our proposed model.

Layer (type)	Output shape	# of parameters
Input layer	(None, 250, 1)	-
Conv1D	(None, 241, 56)	616
BatchNormalization	(None, 241, 56)	224
Dropout	(None, 241, 56)	0
Bidirectional_LSTM	(None, 241, 56)	19,040
LSTM	(None, 241, 28)	9,520
LSTM	(None, 241, 28)	6,384
Global_average_pooling	(None, 28)	-
Dropout	(None, 28)	-
Dense1_for_SBP	(None, 28)	812
Dense2_for_SBP	(None, 16)	464
Dense1_for_DBP	(None, 28)	812
Dense2_for_DBP	(None, 16)	464
Output_for SBP	(None, 1)	17
Output_for DBP	(None, 1)	17
